# Reply to Liu et al., “Inability To Culture Pneumocystis jirovecii”

**DOI:** 10.1128/mBio.01030-18

**Published:** 2018-06-12

**Authors:** Verena Schildgen, Oliver Schildgen

**Affiliations:** a Kliniken der Stadt Köln gGmbH, Klinikum der Privaten Universität Witten-Herdecke mit Sitz in Köln, Institut für Pathologie, Cologne, Germany; Albert Einstein College of Medicine

**Keywords:** PCP, pneumocystis, cell culture

## REPLY

Although we regret that Liu and coworkers were not able to cultivate Pneumocystis jirovecii in our cell culture model proposed 4 years ago (1), we appreciate their comments, as they exactly reflect the problems with which we were dealing. While we presented those attempts that were successful, there were also BAL specimens with proven high mitochondrial large-subunit (mtLSU) rRNA that failed, a fact for which we had no explanation at that time. In our paper, we suggested that quantification based on the established methods may be misleading with regard to the amount of pathogenic particles, and in addition, there are indications that there are differences in P. jirovecii “positivity” concerning life cycle or metabolic stages (2; C. M. Dunaiski et al., submitted); we do not know how they react under culturing conditions. For this reason, 10 BAL specimens is a limited number to establish and check the system, especially as one has to take into account that the CuFi-8 model in its presented form is not yet appropriate for routine culturing. Our published culture system was a prototype. We have tried to acquire funding from both national and international sources to optimize this culture system but were unable to obtain funding to refine and simplify the model developed in 2014.

Unfortunately, Liu et al. do not give any information as to whether the patients received any antibiotic treatment in advance of BAL sampling, which might influence the viability of P. jirovecii, especially in the cohort with low organism loads (3). Moreover, any residual amount of antibiotics might influence the subsequent culture attempts; thus, also the CuFi-8 filters have to be prepared using media without antibiotics. At this point, we apologize for not having explicitly stated this, but we assumed that everyone working with an antibiotic-sensitive organism is aware of this difficulty. This may be one reason why the two high-titer isolates, which remained detectable at remarkable levels in the air-liquid interface cultures, did not further propagate. Furthermore, it has to be taken into account that BAL specimens contain not only pathogens but also cells and cytokines, which may influence the reproducibility of P. jirovecii. Especially in colonized patients with low P. jirovecii levels, it may appear that components of the BAL fluid are still able to control the replication and growth even in cell culture. These speculations together with the findings of inconsistent mitochondrial/nuclear gene ratios and the consequent problem of an appropriate quantification show that it is more than necessary to further improve P. jirovecii culturing.
